# Hypertonic sodium lactate improves microcirculation, cardiac function, and inflammation in a rat model of sepsis

**DOI:** 10.1186/s13054-020-03083-2

**Published:** 2020-06-16

**Authors:** Emmanuel Besnier, David Coquerel, Geoffrey Kouadri, Thomas Clavier, Raphael Favory, Thibault Duburcq, Olivier Lesur, Soumeya Bekri, Vincent Richard, Paul Mulder, Fabienne Tamion

**Affiliations:** 1 Normandie Université, UNIROUEN, Inserm U1096, FHU-REMOD-VHF, 76000 Rouen, France; 2 Department of Anesthesia and Critical Care, Rouen University Hospital, Rouen, France; 3 Division of Intensive Care Units, Centre de Recherche Clinique du CHUS, Université de Sherbrooke, Sherbrooke, Canada; 4 Intensive Care Unit, Lille University Hospital, Lille, France; 5 LIRIC Inserm U995 Glycation: From Inflammation to Aging, Lille, France; 6 Pharmacology Institute of Sherbrooke, Centre de Recherche Clinique du CHUS Université de Sherbrooke, Sherbrooke, Canada; 7 Institute of Clinical Biology, Rouen University Hospital, Rouen, France; 8 Medical Intensive Care Unit, Rouen University Hospital, Rouen, France

**Keywords:** Sodium lactate, Sepsis, Metabolism, Microcirculation, Heart failure

## Abstract

**Background:**

Hypertonic sodium lactate (HSL) may be of interest during inflammation. We aimed to evaluate its effects during experimental sepsis in rats (cecal ligation and puncture (CLP)).

**Methods:**

Three groups were analyzed (*n* = 10/group): sham, CLP-NaCl 0.9%, and CLP-HSL (2.5 mL/kg/h of fluids for 18 h after CLP). Mesenteric microcirculation, echocardiography, cytokines, and biochemical parameters were evaluated. Two additional experiments were performed for capillary leakage (Evans blue, *n* = 5/group) and cardiac hemodynamics (*n* = 7/group).

**Results:**

HSL improved mesenteric microcirculation (CLP-HSL 736 [407–879] vs. CLP-NaCl 241 [209–391] UI/pixel, *p* = 0.0006), cardiac output (0.34 [0.28–0.43] vs. 0.14 [0.10–0.18] mL/min/g, *p* < 0.0001), and left ventricular fractional shortening (55 [46–73] vs. 39 [33–52] %, *p* = 0.009). HSL also raised dP/dt_max_ slope (6.3 [3.3–12.1] vs. 2.7 [2.0–3.9] 10^3^ mmHg/s, *p* = 0.04), lowered left ventricular end-diastolic pressure-volume relation (1.9 [1.1–2.3] vs. 3.0 [2.2–3.7] RVU/mmHg, *p* = 0.005), and reduced Evans blue diffusion in the gut (37 [31–43] vs. 113 [63–142], *p* = 0.03), the lung (108 [82–174] vs. 273 [222–445], *p* = 0.006), and the liver (24 [14–37] vs. 70 [50–89] ng EB/mg, *p* = 0.04). Lactate and 3-hydroxybutyrate were higher in CLP-HSL (6.03 [3.08–10.30] vs. 3.19 [2.42–5.11] mmol/L, *p* = 0.04; 400 [174–626] vs. 189 [130–301] μmol/L, *p* = 0.03). Plasma cytokines were reduced in HSL (IL-1β, 172 [119–446] vs. 928 [245–1470] pg/mL, *p* = 0.004; TNFα, 17.9 [12.5–50.3] vs. 53.9 [30.8–85.6] pg/mL, *p* = 0.005; IL-10, 352 [267–912] vs. 905 [723–1243] pg/mL) as well as plasma VEGF-A (198 [185–250] vs. 261 [250–269] pg/mL, *p* = 0.009).

**Conclusions:**

Hypertonic sodium lactate fluid protects against cardiac dysfunction, mesenteric microcirculation alteration, and capillary leakage during sepsis and simultaneously reduces inflammation and enhances ketone bodies.

## Introduction

Sepsis is a major public health issue responsible for about 6 million deaths per year worldwide [1]. Fluid infusion is essential to maintain cardiac preload and therefore end-organ perfusion and oxygenation. However, excessive fluid therapy may lead to a positive fluid balance associated with mortality during septic shock [2, 3]. Moreover, intravenous fluids may augment septic endothelial dysfunction, potentially negating the beneficial hemodynamic effects of fluid resuscitation [4]. The nature of the administrated fluid is currently the focus of extensive literature [5]. Indeed, 0.9% sodium chloride (NaCl) is widely used but may lead to hyperchloremia and metabolic acidosis, but also a reduction in renal glomerular filtration [6, 7]. Moreover, an experimental study suggested pro-inflammatory effects of hyperchloremic acidosis [8]. “Balanced fluids,” containing a low chloride concentration, may limit these negative effects and result in fewer use of renal replacement therapy or persistent renal dysfunction [9]. Lactate-containing fluids may also be of interest because of the absence of chloride and may provide merits in critically ill patients. Moreover, the lactate molecule itself may provide energy supply through its oxidation [10]. Energy crisis during sepsis participates in organ failure. Indeed, several metabolic pathways are disturbed: alteration of mitochondrial function, resistance to insulin, and defect in β-oxidation [11–13]. Several data argue that lactate is a major metabolite during inflammation and represents an important source of energy for various organs, including the heart [14, 15], raising its interest during critical illness. Some experimental and human studies observed beneficial effects of molar hypertonic sodium lactate (HSL) in various settings, such as brain injury, cardiac dysfunction, and even human Dengue infection, with a notable effect on microcirculation [16–19]. Moreover, its use during endotoxemia induced an improvement of microcirculation and fluid balance [20, 21] but was not explored during sepsis. We therefore hypothesize that HSL may improve microcirculation during sepsis, and we explored its effects in a rat model of sepsis.

## Material and methods

We realized a prospective, randomized, controlled experimental study approved by the Ethics Committee for Animal Research (*CENOMEXA no. 54*, approval number #8093-2016112515181383, 2 April 2019). All procedures were performed in accordance with the French Ethics Committee, the guidelines of the European Parliament directive 2010/63/EU, and the Council for the Protection of Animals Used for Scientific Purposes. The elaboration of this manuscript adheres to the ARRIVE guidelines. The experimental procedures are extensively detailed in Additional file 1.

### Animal procedures

The primary objective was to compare HSL versus 0.9% NaCl on microcirculation. Based on previous data [21], we a priori calculated a number of 10 male Sprague-Dawley rats (400–500 g) per group to be sufficient to identify a difference of 20% in microcirculatory flow with a 5% α-risk and a 90% power. Secondary objectives were cardiac function, capillary leakage, inflammation, and metabolism. Some of these objectives required additional animals because of incompatibility with the microcirculation study. Based on our previous experience, 5 animals per group were included for Evans blue and 7 animals per group for pressure-volume loops.

### Experimental protocol

Sepsis was induced by cecal ligation and puncture (CLP). Animals were anesthetized by intraperitoneal injection of ketamine/xylazine (75/5 mg/kg). A 3-F polyurethane perfusion catheter was inserted in the right jugular vein. The cecum was ligated (75% of its volume) and punctured (16-G needle) to externalize feces. At the end of the procedure, animals were randomized in four groups: CLP-HSL receiving 11.2% sodium lactate (1000 mmol/L of sodium + 1000 mmol/L of lactate, APHP, France), CLP-NaCl receiving 0.9% NaCl (154 mmol/L of chloride + 154 mmol/L of sodium), CLP-HSB receiving 8.4% sodium bicarbonate (1000 mmol/L of bicarbonates + 1000 mmol/L of sodium), and sham with cervicotomy and laparotomy but no catheter/CLP. Unfortunately, only 2 over the 6 first rats in the HSB group survived. For ethical reasons, and in accordance with the legislative authorizations delivered for this study, we did not complete this group. CLP rats did not have access to water and chow and received a 2.5- mL/kg/h infusion of studied fluids for an 18-h period [22]. Eight percent sterile dextrose was added to CLP-NaCl fluid to bring an equivalent amount of calories as in the CLP-HSL group [20]. Rat under experiment was housed alone in a cage. Three different sets of experiments were realized after anesthesia with ketamine/xylazine (75/5 mg/kg): (1) echocardiography and laser speckle imaging (*n* = 10/group), (2) Evans blue assay (*n* = 5/group), or (3) pressure-volume loops (*n* = 7/group).

### Echocardiography

Echocardiography was realized under anesthesia, and left ventricular end-diastolic and end-systolic diameters (LVEDD and LVESD) were measured using M-Mode allowing to calculate left ventricular fractional shortening (LVFS). Pulsed wave Doppler was used for the acquisition and calculation of the mitral E/A ratio. Cardiac output expressed per gram of animal was measured through the proximal pulmonary artery.

### Mesenteric perfusion

After echocardiography, microcirculation acquisition was performed by laser speckle contrast imaging on the gut. Image analysis was realized in four similar regions of interest, distant from the jejunal site, and mean values are expressed as perfusion units (PU).

### Capillary leakage and Evans blue assay

Evans blue was injected intravenously and was flushed away from the bloodstream by paraformaldehyde 45 min later. The heart (left ventricle), lungs, gut, and liver samples were dehydrated at 60 °C for 5 days and then incubated in 10% formamide at 37 °C for 3 days. The absorbance of the centrifugated supernatant was measured using spectrophotometry at 620 nm.

Fluid balance (the difference between urine output and infused fluids) was recorded in the CLP-NaCl and CLP-HSL groups.

### Hemodynamics

Pressure-volume loops and hemodynamic were obtained in the left ventricle (LV). LV end-systolic pressure (LVESP), LV end-diastolic pressure (LVEDP), dP/dt_min_, dP/dt_max_, and LV relaxation constant tau (Weiss Method) were recorded/calculated. LVESP and LVEDP relation (LVESPVR and LVEDPVR) were calculated as indicators of load-independent LV contractile function and LV compliance. A blood sample was taken at the end of the procedure to measure and compare plasma osmolality (mosmol/kg) using a cryoscopic osmometer.

### Biological parameters

At the end of the procedure, a maximal volume of blood was sampled.

#### Inflammation and capillary leakage-related markers

IL-1β, TNFα, and IL-10; vascular endothelial growth factor type A (VEGF-A), and syndecan-1 were measured using ELISA.

#### Biochemistry

Urea, sodium, potassium, and chloride were measured in the blood and urine. Albumin was measured in the blood. The absolute quantity of ions excreted during the experiment was calculated, and the difference between the infused and excreted quantity of ions during the whole experiment was calculated and reflected the body excess of ions over the infusion period.

#### Metabolism

Blood content of glucose, lactate, pyruvate, 3-hydroxybutyrate, and acetoacetate was measured.

### Statistical analysis

The distribution of data was evaluated using a d’Agostino test, and data are presented as medians with interquartiles [IQ1–IQ3] or means with standard deviations (± SD). Comparisons between the groups were realized using a non-parametric test (Kruskal-Wallis or Mann-Whitney) or a parametric test (ANOVA or t-Student) according to the normality of the distribution, and in case of significant results, post hoc analyses were performed using either Dunn’s or Holm-Sidak’s multiple comparisons test. Two comparisons were realized: CLP-NaCl group versus sham and CLP-NaCl group versus CLP-HSL. Because we aimed to evaluate the effects of HSL during sepsis, the comparison between sham and CLP-HSL was not relevant and may reduce the power of the analyses. All comparisons were realized using Prism v8.0 (GraphPad, USA). *p* < 0.05 was considered significant. In case of missing data concerning the secondary endpoints of each set of experiments, the median value of the control group was applied to favor the null hypothesis.

## Results

Over the 22 rats per group (for the 3 sets of experiment), 2 in the CLP-HSL group and 1 in the CLP-NaCl group died before the end of the perfusion (new rats were added in replacement).

### Mesenteric perfusion

**Fig. 1:**
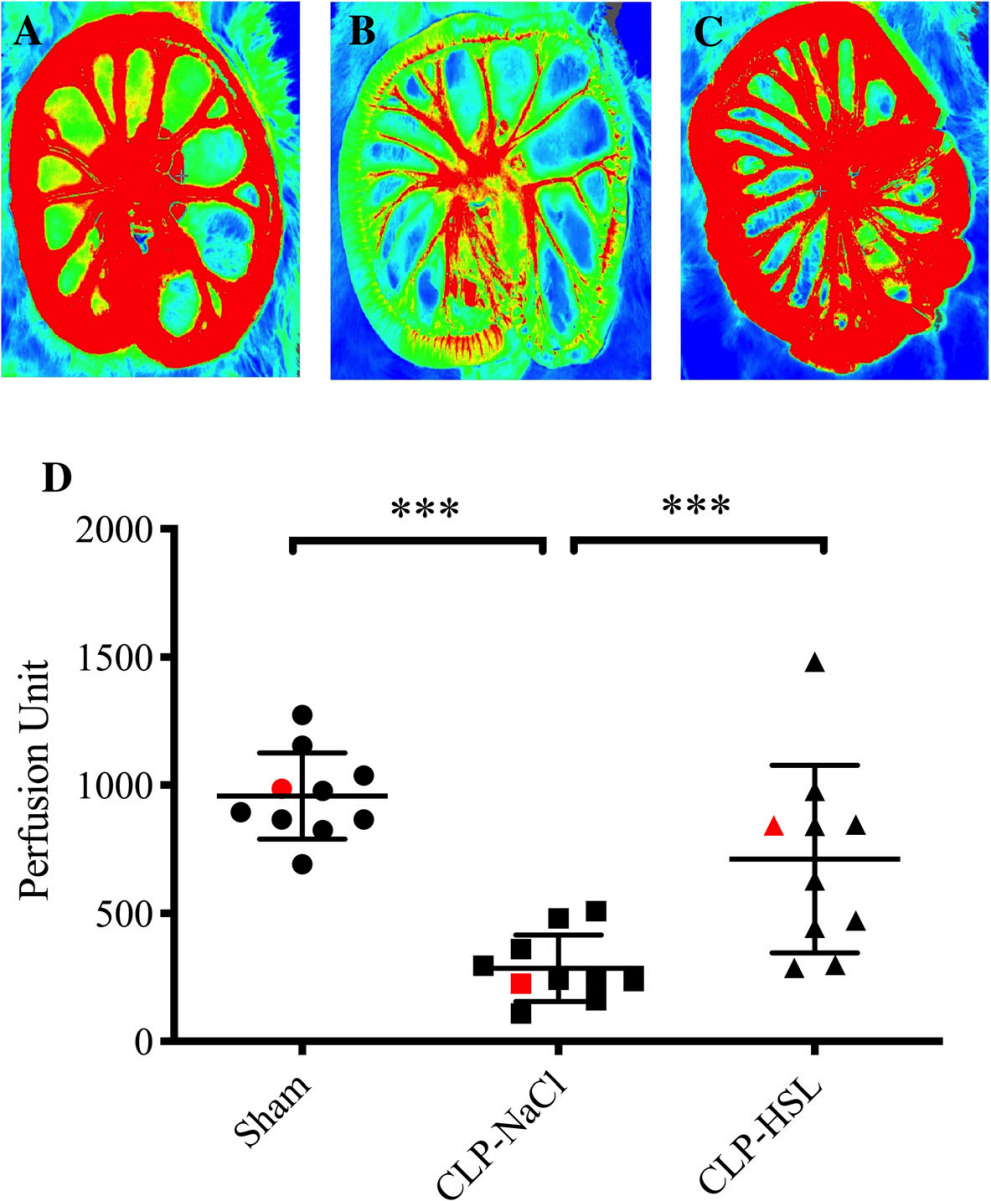
Mesenteric microcirculation evaluated by laser speckle imaging (*n* = 10/group). Three examples are presented. **a** Sham. **b** CLP-NaCl. **c** CLP-HSL. **d** Mean ± SD values of perfusion units in the 3 groups. Red symbols represent the value of the presented examples. ***p < 0.001

The mean value of mesenteric perfusion was markedly reduced in the CLP-NaCl group in comparison with the sham group (286 ± 129 vs. 957 ± 169 PU, *p* < 0.0001). On the contrary, HSL greatly enhanced perfusion in comparison with NaCl in CLP rats (712 ± 366 vs. 286 ± 129 PU, *p* = 0.0006, Fig. 1).

### Capillary leakage

**Fig. 2:**
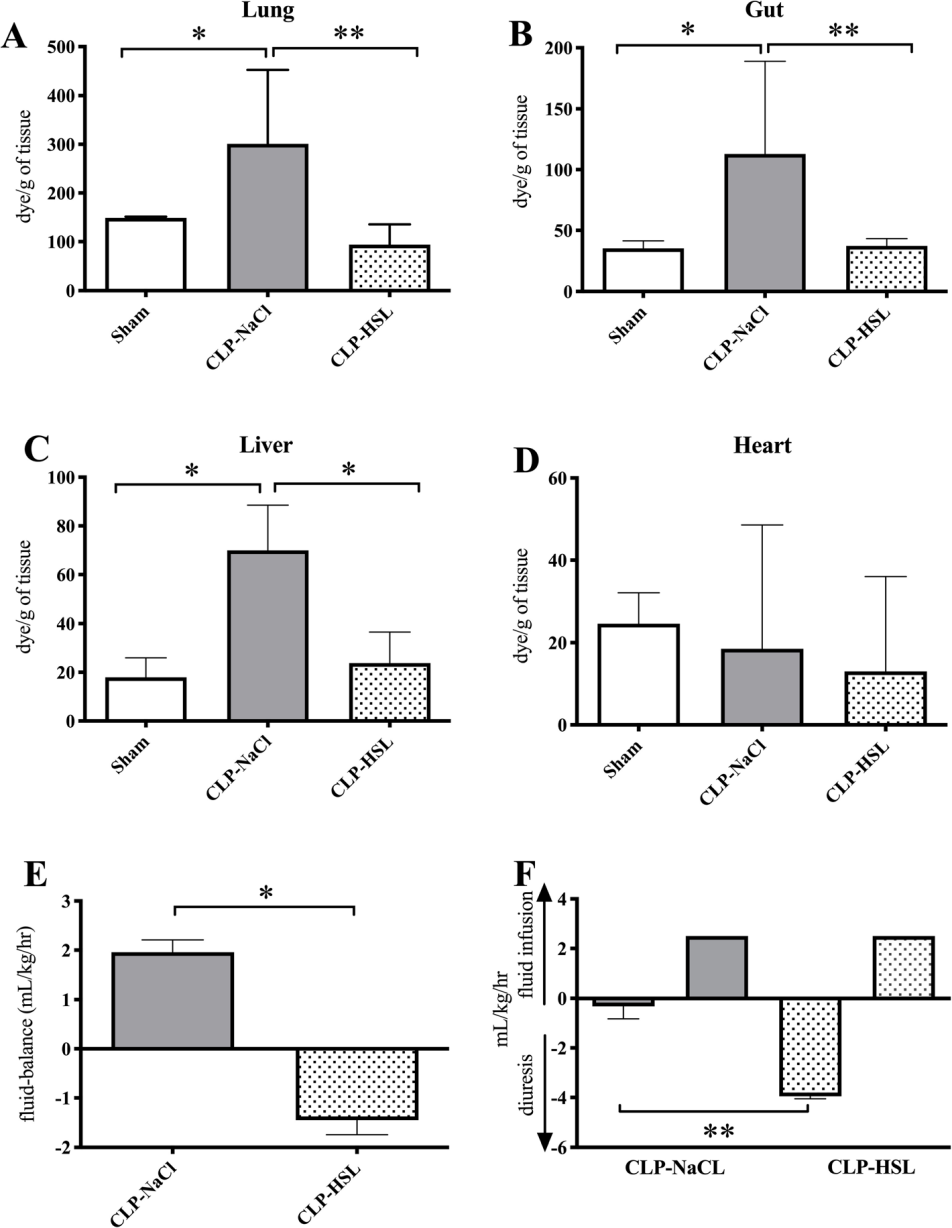
Capillary leakage explored using Evans blue assay (*n* = 5/group) in the Lungs (**a**), gut (**b**), liver (**b**), and heart (**d**). Results for fluid balance (**e**) and urine output and infused fluid (**f**) expressed in mL/kg/h between the CLP-NaCl and CLP-HSL groups.*p < 0.05, **p < 0.01, ***p < 0.001

Evans blue diffusion increased in the CLP-NaCL group compared with the sham group for the lungs (300 [253–452] vs. 149 [83–152], *p* = 0.04), the gut (113 [88–189] vs. 35 [21–41], *p* = 0.009), and the liver (70 [50–89] vs. 18 [15–26], *p* = 0.02) (Fig. 2). No difference was observed for the heart. Conversely, in CLP rats, HSL resulted in a reduction of Evans blue diffusion in comparison with NaCl for the lungs (94 [78–136] vs. 300 [253–452], *p* = 0.006), the gut (37 [31–43] vs. 113 [88–189], *p* = 0.03), and the liver (24 [14–37] vs. 70 [50–89], *p* = 0.04), but not for the heart. Similarly, the fluid balance was drastically improved by HSL versus NaCl (− 1.5 [− 1.7 to − 0.7] vs. 2.0 [1.6–2.2] mL/kg/h, *p* = 0.002) with a significantly higher urine output in HSL rats (3.9 [3.2–4.1] vs. 0.3 [0.3–0.8] mL/kg/h, *p* = 0.008) (fluid infusion was 2.5 mL/kg/h in both groups as presented in the “Material and methods” section).

### Echocardiography

**Fig. 3:**
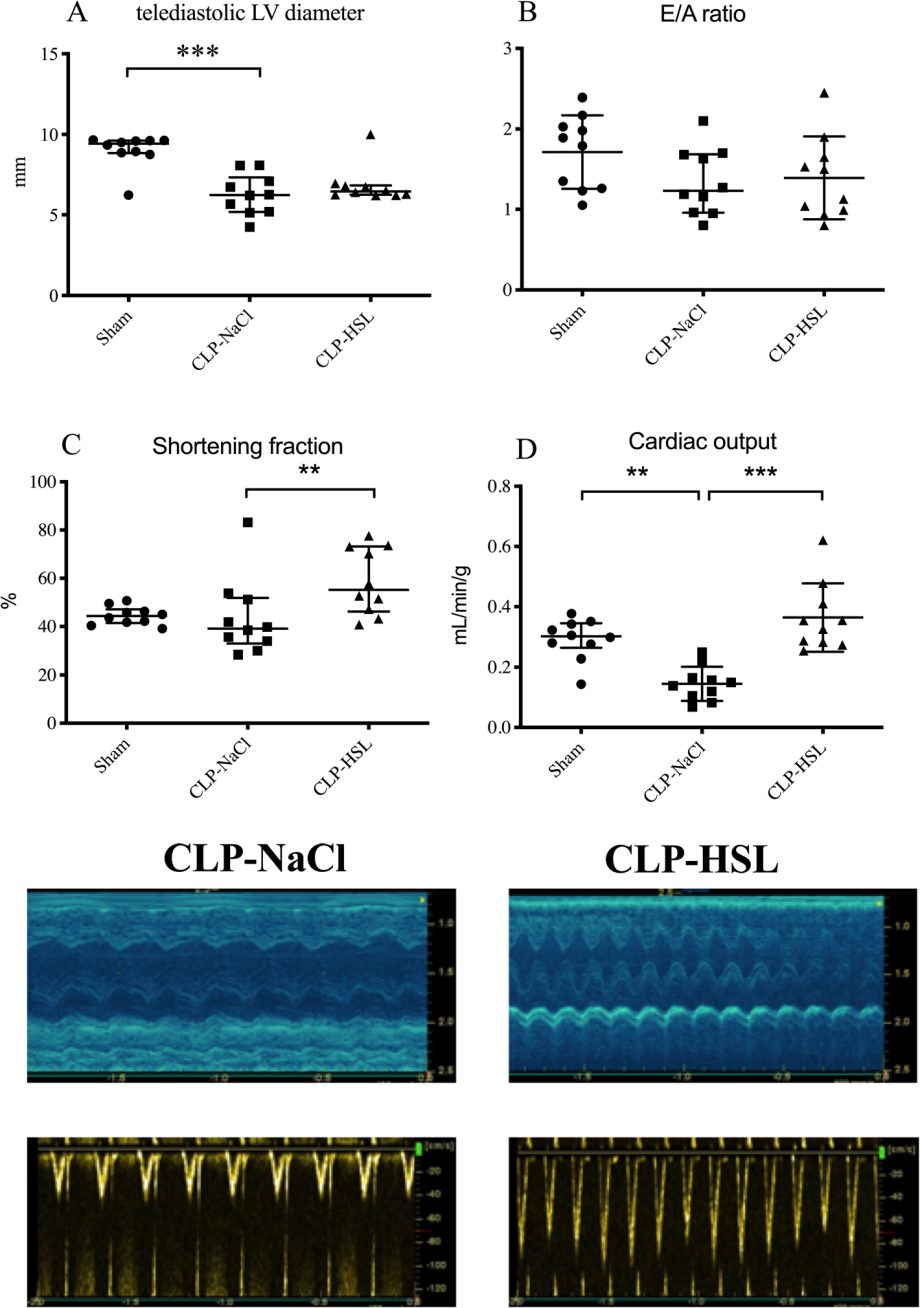
Trans-thoracic echocardiography (*n* = 10/group). **a** End-diastolic left ventricular diameter. **b** E peak velocity/A peak velocity ratio of the mitral flow. **c** Shortening fraction evaluated by time-movement mode. **d** Cardiac output. Examples are presented in the lower part of the figure for the shortening fraction (M-Mode) and velocity-time integral (pulsed wave Doppler). **p < 0.01, ***p < 0.001

The CLP-NaCl group presented a reduced cardiac output in comparison with sham (0.14 [0.01–0.18] vs. 0.30 [0.26–0.34] mL/min/g, *p* = 0.004) despite the absence of difference for LV fractional shortening (39 [33–52] vs. 44 [41–47] %, *p* = 0.9) (Fig. 3). Concerning the preload static indices available in our model, LVEDD was reduced in the CLP-NaCl group versus sham (6.2 [5.2–7.3] vs. 9.4 [8.8–9.6] mm, *p* = 0.001), but no difference was observed concerning the E/A mitral flow ratio (1.3 ± 0.4 vs. 1.7 ± 0.5, *p* = 0.2). Left ventricular systolic function was greatly improved by HSL vs. NaCl in CLP rats with higher cardiac output (0.34 [0.28–0.43] vs. 0.14 [0.01–0.18] mL/min/g, *p* < 0.0001) and shortening fraction (55 [46–73] vs. 39 [33–52] %, *p* = 0.009). No difference concerning the preload static indices was observed (LVEDD, 6.5 [6.3–6.8] vs. 6.2 [5.2–7.3] mm, *p* = 0.6; E/A ratio, 1.4 ± 0.5 vs. 1.3 ± 0.4, *p* = 0.8).

### LV pressure-volume loops

The results are presented in Table 1. In comparison with sham, CLP-NaCl presented reduced mean, diastolic, systolic, and pulse arterial pressures. Pressure tracings showed a reduction in dP/dt_max_ and LVESP, resulting from a reduction in ventricular inotropism. Nevertheless, LVESPVR was not different, suggesting that the alteration of inotropism may be related to an alteration of cardiac pre- and/or afterload. On the contrary, dP/dt_min_ and LVEDPVR were modified in CLP-NaCl rats, suggesting an alteration of ventricular compliance, independent from the cardiac preload.

**Table 1:** Invasive hemodynamic parameters and pressure-volume loops (*n* = 7/group)

	Sham	CLP-NaCl	CLP-HSL	*p* value	
				NaCl vs. sham	HSL vs. NaCl
Pulse pressure (mmHg)	45 [33–48]	22 [19–32]	35 [30–37]	0.006	0.2
Systolic blood pressure (mmHg)	120 ± 19	73 ± 23	88 ± 32	0.006	0.5
Diastolic blood pressure (mmHg)	79 ± 13	50 ± 20	55 ± 29	0.03	0.8
LVEDP (mmHg)	2.3 [1.2–4.4]	2.6 [1.7–4.4]	2.8 [1.9–3.6]	0.9	1
LVESP (mmHg)	114 ± 21	68 ± 22	87 ± 32	0.006	0.3
dP/dt_max_ (mmHg/s)	7997 ± 2347	2974 ± 988	7110 ± 4582	0.01	0.04
dP/dt_min_ (mmHg/s)	6492 ± 2045	3079 ± 1503	5489 ± 3564	0.04	0.2
LVESPVR	22 ± 3	21 ± 13	20 ± 3	ns	
LVEDPVR	1.0 ± 0.3	3.2 ± 1.2	1.6 ± 0.8	0.0002	0.005
Tau	8.2 ± 3.1	10.6 ± 1.8	9.5 ± 3.6	ns	
Osmolality (mosmol/kg)	311 [308–317]	316 [311–323]	318 [317–330]	0.8	0.2

Results are presented as means ± standard deviations or medians with the first and third quartiles according to the normality of the population. Only the results of the post-test are presented as numerical data. In case of a non-significant primary test, the results are presented as “ns”

*LVEDP* left ventricular end-diastolic pressure, *LVESP* left ventricular end-systolic pressure, *LVEDPVR* left ventricular end-diastolic pressure-volume relationship, *LVESPVR* left ventricular end-systolic pressure-volume relationship, *Tau* isovolumic relaxation constant, *NaCl* sodium chloride, *HSL* hypertonic sodium lactate

The administration of HSL did not result in different levels of arterial blood pressure versus NaCl, but a higher dP/dt_max_ and no difference in LVESPVR, suggesting an improvement in inotropism but possibly related to an increase in preload. Reduction in LVEDPVR suggested an improvement in ventricular compliance independently from the cardiac preload/afterload.

Plasma osmolality did not vary between CLP-NaCl versus sham rats and CLP-NaCl versus CLP-HSL rats in this experiment (Table 1).

### Biological parameters

The results are presented in Table 2. In CLP rats, infusion of HSL was associated with a significant rise in sodium plasma concentration, and in sodium urinary excretion, while plasma chloride and potassium were reduced. ∆Na^+^, representing the body accumulation of sodium over the infusion period, was not significantly different between the CLP-HSL and CLP-NaCl groups.

**Table 2:** Biological parameters

	Sham	CLP-NaCl	CLP-HSL	*p* value	
				Sham vs. NaCl	HSL vs. NaCl
Blood ionogram (*n* = 6)					
Na^+^ (mmol/L)	138 [138–143]	141 [140–143]	153 [150–157]	1	0.02
K^+^ (mmol/L)	4.8 [4.5–5.0]	4.7 [4.2–5.2]	3.7 [3.1–4.0]	1	0.02
Cl^−^ (mmol/L)	98 [96–99]	100 [99–101]	97 [94–99]	1	0.02
Urea (mmol/L)	6.9 [5.7–8.3]	16.2 [11.3–24.6]	9.6 [8.5–10.7]	< 0.0001	0.04
Urine ionogram (*n* = 6)					
Na^+^ (mmol/L)	149 [58–163]	26 [21–39]	441 [362–509]	0.2	0.0004
Na^+^ (mmol/day)	2.5 [2.1–2.8]	0.1 [0.1–0.1]	9.9 [8.2–12.5]	0.1	0.0003
∆Na^+^ (mmol)		2.4 [2.4–2.8]	4.9 [2.7–6.2]		0.06
K^+^ (mmol/L)	254 [196–282]	325 [295–406]	104 [88–142]	0.2	0.0005
K^+^ (mmol/day)	4.6 [3.9–4.8]	1.3 [0.7–2.2]	2.6 [2.0–2.9]	0.001	0.3
∆K^+^ (mmol)		− 1.3 [0.7–2.2]	− 2.6 [2.0–2.9]		
Cl^−^ (mmol/L)	185 [143–231]	89 [67–126]	107 [85–137]	0.02	0.8
Cl^−^ (mmol/day)	3.4 [2.9–3.7]	0.3 [0.1–0.8]	2.5 [1.9–3.2]	0.002	0.03
∆Cl^−^ (mmol)		2.2 [2.0–2.4]	− 2.5 [1.9–3.2]		0.002
Blood metabolic parameters (*n* = 10)					
Glucose (mmol/L, *n* = 10)	10.5 ± 2.4	4.9 ± 1.1	9.4 ± 4.5	0.0006	0.002
Lactate (mmol/L)	1.6 ± 1.0	3.5 ± 1.5	6.7 ± 4.6	0.1	0.04
Pyruvate (μmol/L)	32 ± 22	122 ± 51	249 ± 186	0.09	0.03
Lactate/pyruvate ratio	51 [34–97]	28 [17–37]	24 [17–31]	0.06	1
3-Hydroxybutyrate (μmol/L)	514 ± 249	212 ± 105	445 ± 303	0.02	0.03
Acetoacetate (μmol/L)	317 ± 198	149 ± 77	216 ± 137	ns	
Blood inflammation and capillary leakage parameters (*n* = 10)					
IL-1β (pg/mL)	48 ± 11	993 ± 851	272 ± 215	0.0006	0.004
TNFα (pg/mL)	6 ± 2	59 ± 31	28 ± 23	< 0.0001	0.005
IL-10 (pg/mL)	207 ± 29	1015 ± 389	601 ± 523	0.0001	0.02
VEGF-A (pg/mL)	240 [226–280]	261 [250–287]	198 [185–250]	0.4	0.009
Syndecan-1 (pg/mL)	8.5 ± 4.0	9.7 ± 2.6	7.7 ± 2.0	ns	
Albumin (g/L)	25.6 ± 2.2	22.2 ± 2.2	23 ± 2.6	0.001	0.4

∆ion = ion administrated (mmol) − ion excreted (mmol)

*NaCl* sodium chloride, *HSL* hypertonic sodium lactate

Urea was twofold higher in CLP-NaCl compared to sham, whereas HSL resulted in lower plasma levels.

Albumin was reduced in CLP-NaCl versus sham but not versus CLP-HSL.

Concerning metabolism, the CLP-NaCl group presented a twofold reduction in glycemia versus sham with a reduction in plasma 3-hydroxybutyrate. Conversely, HSL was associated with higher plasma levels of glucose, lactate, pyruvate, and 3-hydroxybutyrate in comparison with NaCl, but no difference was observed between the groups for lactate/pyruvate ratio.

### Inflammation

**Fig. 4:**
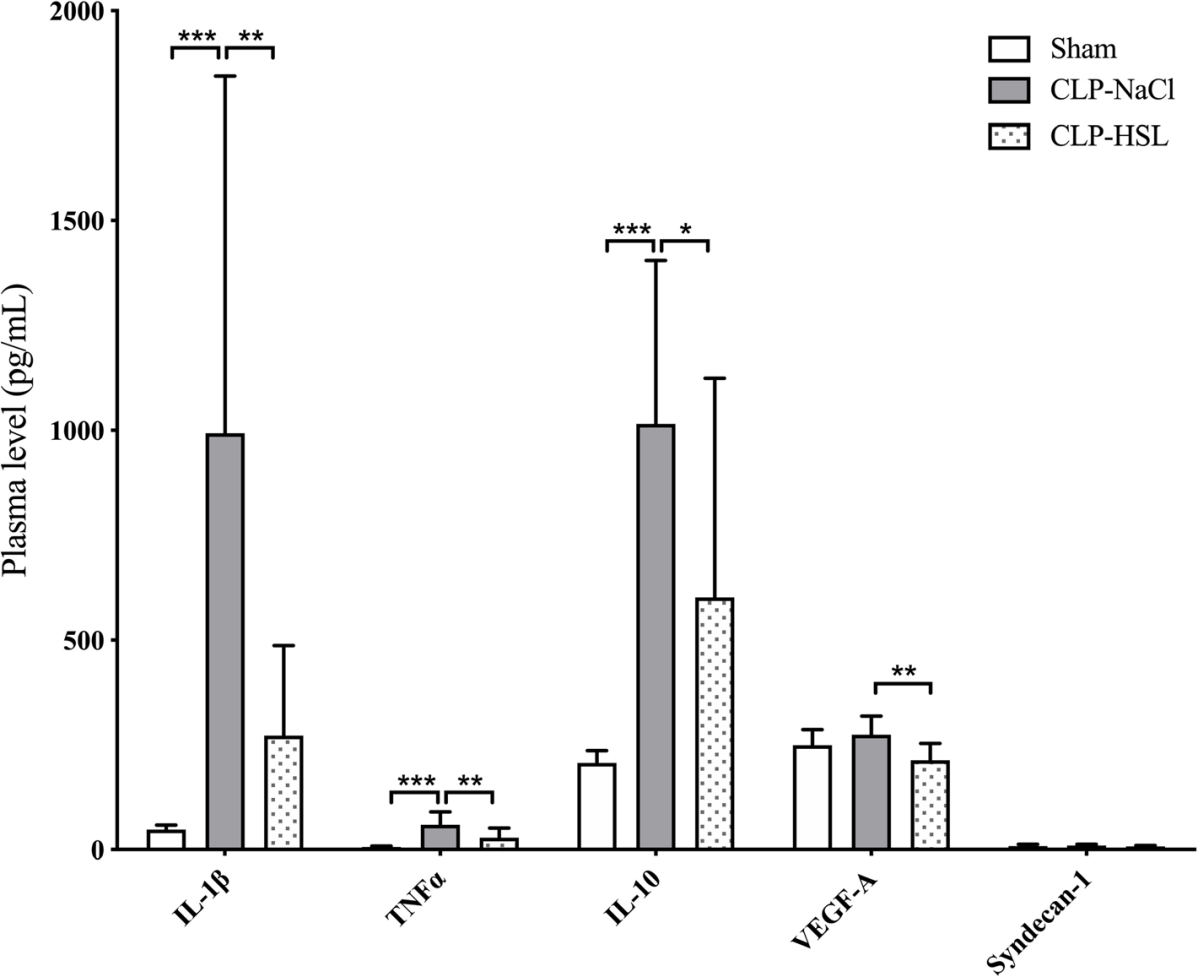
Plasma levels of cytokines, VEGF-A, and syndecan-1. *p < 0.05, **p < 0.01, ***p < 0.001

The CLP-NaCl group presented an enhanced inflammation in comparison with sham with an important rise in cytokines IL-1β, TNFα, and IL-10 (Fig. 4, Table 2). HSL reduced plasma levels of IL-1β, TNFα, and IL-10 versus NaCl.

No difference was observed for VEGF-A or syndecan-1 between the sham, CLP-NaCl, and CLP-HSL groups except a reduction in VEGF-A levels between CLP-NaCl and CLP-HSL groups.

## Discussion

Our study highlights the beneficial effects of HSL during sepsis. This brings new insights for its use during critical illness.

### HSL modulation of CLP-induced microcirculation alteration

HSL greatly improved microcirculation with improved tissue perfusion, capillary leakage, and fluid balance. Endothelial *adherens* junctions and glycocalyx are key elements for the regulation of fluid movements [23–25]. VEGF-A can modulate *adherens* junctions through the endocytosis of the adhesion molecule VE-cadherin [26], resulting in an enlargement of the intercellular gap and therefore fluid shift to the interstitium, and have been described to be associated with the severity of sepsis [27]. Our results highlight a reduction in the circulating levels of VEGF-A in the HSL group. Nevertheless, many other factors are implicated in the regulation of vascular permeability, such as Angiopoietin-2, Slit/Robo4, or heparin-binding protein [25], and may explain the observed discrepancy between an important improvement in capillary leakage and a small difference in VEGF-A levels. Despite several publications described a degradation of glycocalyx during sepsis [24], we did not observe a difference for syndecan-1. Nevertheless, our model focused on the early phase of sepsis (< 24 h) whereas the increase of syndecan-1 has been described to be elevated at day 2 in septic patients, but not at admission, suggesting a time-dependent variation of this marker [28, 29]. Moreover, septic inflammation may activate many proteolytic processes such as metalloproteinases, heparanase, and hyaluronidase, leading to vascular hyper-permeability through the degradation of many other compounds of the glycocalyx. Indeed, we demonstrated in our model that inflammation is deeply reduced by HSL. This may be explained by the non-specific improvement in various functions (cardiac, microcirculation, renal), but also by a specific effect on inflammation pathways. Lactate may act through the G protein-coupled receptor GPR81, which senses both lactate and hydroxybutyrate and is widely distributed within the organism, notably in endothelial cells [30]. This receptor has been demonstrated to modulate the production of IL-1β, IL-6, and IL-12 [31, 32]. Thus, regulation of inflammatory process by HSL may be a cornerstone of the improvement in microcirculation. One can argue that change in plasma levels of the various markers may be due to the difference in vascular volume, but the absence of difference in albumin blood content and plasma osmolality did not favor this hypothesis.

### Cardiac function

HSL improved myocardial efficiency as evidenced by enhanced LVEF, cardiac output, and systolic dp/dt_max_ slope. The improvement in preload may be partly responsible for these changes, since left ventricular end-diastolic pressure-volume relation was similar in the septic groups. However, it is likely that the contribution of volemia may be limited because of an absence of difference in osmolality between the groups. This absence of difference in plasma tonicity may be secondary to an enhanced urinary excretion of Na^+^, resulting in the absence of difference concerning global sodium accumulation (∆Na^+^). Moreover, albumin blood content and cardiac indices E/A ratio and LVEDD, as well as blood pressure, were also similar between the groups. Our results also showed a preload-independent improvement in cardiac compliance—diastolic function, as observed by a reduction in the left ventricular end-diastolic pressure-volume relation.

The improvement of cardiac function by HSL had been demonstrated in non-septic situations, even compared to a hypertonic control group [20, 33]. In our model, the intense modulation of inflammation by HSL may have protected the heart from injuries because pro-inflammatory cytokines mediate sepsis-induced cardiac dysfunction [34]. Moreover, it has been described that the heart changes its metabolic substrate during shock, shifting from free fatty acids to lactate oxidation [35, 36], and our study reinforces this idea. Our data, in addition to these previous studies, support the hypothesis of a direct effect of lactate-containing fluid on cardiac function rather than an osmotic effect.

### Renal function

HSL improved renal function with an increase in diuresis and a reduction in urea. HSL has been previously described as beneficial on kidney function during experimental endotoxemia and protected animals from thrombosis of the glomerular capillaries [21, 37]. HSL contains no chloride and reduced both chloride blood content and ∆Cl^−^ whereas this ion is highly detrimental to the kidney.

### Metabolism

Lactate is a major element of bioenergy, at the crossroad of many energetic pathways. It can directly produce ATP through its oxidation via pyruvate transformation and the mitochondrial citric acid cycle, and it can promote the production of other energy substrates such as ketone bodies, or even glucose through neoglucogenesis. Contrary to glucose, its transport into cells and mitochondria is regulated by a membrane transporter independent from insulin [10]. Sepsis induces resistance to insulin resulting in little glucose availability for tissues [38]. We demonstrated that HSL raised both lactate and pyruvate levels with preserved lactate/pyruvate ratio, suggesting the utilization of this metabolite whereas the deprivation of circulating lactate levels has been described as detrimental for the septic heart [36]. Moreover the diminution in K^+^ blood content in the HSL group, surrogate of a probable blood alkalinization, is another argument for lactate utilization. Ketone bodies 3-hydroxybutyrate can be produced from pyruvate-derived acetyl-CoA in various organs. Then, its shuttle to the heart may provide ATP through its secondary integration in the citric acid cycle after reconversion to acetyl-CoA. Its administration in patients with chronic heart insufficiency has been demonstrated to improve cardiac systolic function [39]. We demonstrated that HSL rises lactate and pyruvate but also 3-hydroxybutyrate blood levels. Thus, the observed improvement in cardiac function may also be secondary to this rise of ketones, because caloric intakes in both CLP-NaCl and CLP-HSL groups were similar. We also demonstrated that HSL enhanced glucose blood content, despite the fact that the NaCl group received an additional infusion of dextrose suggesting increased neoglucogenesis from lactate or/and a sparring effect of glucose utilization because of the oxidation of lactate. Only one study previously explored half-molar sodium lactate in an ovine model of sepsis with deleterious effects (increased severity of shock, hypoxemia, and mortality) [40]. This study presented several limits. First, the ovine model is far less studied than rat or mouse models, and this limits the transposition to humans. Second, the lactate group received 30% less fluids than the hypertonic control group. But the main difference is a possible absence of oxidation of the perfused lactate with an increased lactate/pyruvate ratio and an absence of difference in glucose blood content, suggesting both an absence of oxidation and shuttle of lactate in this model, contrary to ours.

### Strengths and limits

Our study has several limitations. The main one is the absence of a hypertonic control group because of the high mortality rate in the “hypertonic bicarbonates” group. The reasons are unclear. Nevertheless, the use of HSL was previously associated with better microcirculation, inflammation, and renal function during endotoxemia in comparison with HSB [20, 21], making it less interesting in our work to carry out a hypertonic control group. The use of a hypertonic NaCl group as a control has not been realized as we believe that the comparison with HSL is not suitable. Although hypertonic NaCl may be of interest in reducing the volume of fluid administrated during resuscitation, and may have interesting hemodynamic effects, this fluid is unbalanced and therefore presents a major chloride content. A high chloride intake is potentially responsible for hyperchloremic acidosis, impaired renal function, and even inflammatory process and could even be associated with mortality in ICU patients [6–8, 41, 42]. Moreover, a meta-analysis of the few clinical trials available did not show any difference in major outcomes with the use of hypertonic saline during sepsis [43]. We also discussed above that tonicity between 0.9% NaCl and HSL groups appeared similar and may not explain the beneficial effects of HSL. In addition, the in vivo tonicity of HSL is difficult to estimate due to the rapid metabolism of lactate (which accounts for half of the in vitro tonicity) and the major natriuresis after HSL limiting the pro-osmotic effect of sodium. Thus, the constitution of a hypertonic control group is highly hazardous because of the unknown actual tonicity of infused HSL (because of metabolization of lactate), and this control group may have a higher in vivo tonicity despite similar in vitro tonicity, which may have explained the over-mortality in the HSB group. Finally, we evoked the specific effect of HSL through its GPR81 receptor, but this hypothesis needs further explorations. Finally, and as always, the transposition of rodent models to humans is questionable. Nevertheless, the use of a septic model in rats allowed us to explore many aspects of organ dysfunction in a standardized model, with a robust methodology.

## Conclusion

In conclusion, the use of hypertonic lactate-containing fluid in an experimental sepsis model improved microcirculation, capillary leakage, and cardiac and renal functions, potentially through metabolic and inflammatory pathways. Both experimental and human studies are warranted to deepen the mechanisms of sepsis improvement by HSL and the possible transposition to clinical sepsis.

## Supplementary information

**Additional file 1.** : Detailed experimental procedures.

## Data Availability

The datasets used and/or analyzed during the current study are available from the corresponding author on reasonable request.

## Abbreviations

ATP: Adenosine triphosphate
CLP: Cecal ligation and puncture
HSB: Hypertonic sodium bicarbonate
HSL: Hypertonic sodium lactate
LV: Left ventricle
LVED: Left ventricular end-diastolic diameter
LVEDP: Left ventricular end-diastolic pressure
LVEDPR: Left ventricular end-diastolic pressure relation
LVESP: Left ventricular end-systolic pressure
LVESPVR: Left ventricular end-systolic pressure relation
LVSF: Left ventricular shortening fraction
IL: Interleukin
PU: Perfusion units
TNFα: Tumor necrosis factor α
VEGF: Vascular endothelial growth factor
