# Supplementary material for: Hypertonic sodium lactate improves microcirculation, cardiac function, and inflammation in a rat model of sepsis
Source: Crit Care. 2020 Jun 16;24:354. doi: 10.1186/s13054-020-03083-2 (PMC7298868; doi:10.1186/s13054-020-03083-2)
Supplement: Supplementary file 1 — Additional file 1. : Detailed experimental procedures. [file 13054_2020_3083_MOESM1_ESM.docx]

Supplementary File 1: detailed experimental procedures

Caecal ligature and puncture model

Male Sprague-Dawley rats, weighting 400-500g (Janvier Labs, France), were housed at a constant temperature (21°C) with 14 h/10 h light/dark cycle (2 rats per cage before experiment, then 1 per cage during experiment). All animals had free access to standard chow and drinking tap water until the beginning of the study. The model of sepsis consisted in a polymicrobial peritonitis by caecal ligation and puncture (CLP). The day of the experiment, animals were randomized to one of the groups using sealed envelopes. They were anesthetized by intraperitoneal injection of ketamine/xylazine (75/5 mg/kg). After skin sterilization (alcoholic-povidone-iodine), a cervicotomy was performed to insert a 3F polyurethane perfusion catheter (Instech Laboratories, USA) in the right external jugular vein under magnifying glasses. The catheter was tunnelled under skin and connected to a perfusion harness (Instech Laboratories, USA) and then skin was closed using 2.0 vicryl (Ethicon, USA). A median laparotomy was then performed to expose the caecum. After gently ligation of 75% of its volume, a 16G puncture was performed in the antimesenteric side to externalize feces in the peritoneal cavity. Muscular facia and skin were sutured using 2.0 vicryl (Ethicon, USA). Analgesia was performed using topical 1% lidocaine on muscular and cutaneous wounds and subcutaneous injection of tramadol (20 mL/kg). In case of pain, observed using a standardized scale specific for rodents, an additional dose of tramadol (SC 20 mL/kg) was administrated every 8 hours. One mL of 0.9% NaCl was i.v injected at the end of procedure to compensate the blood loss. This model of sepsis has been observed to produce a 60% mortality at 48 hrs (data not published).

Except for the sham group, rats in CLP-HSL and CLP-NaCl groups received a 2.5 mL/kg/h infusion of fluids for a 18 h period using a single syringe infusion pump (KDS100, KDScientific, USA). Because lactate provides 0.32 kcal/mL, CLP-NaCl group received an equivalent number of calories by the addition of 0.087g/mL of dextrose (8% concentration, 1g of dextrose = 4 kcal) in the perfused fluid. To ensure the sterility of the solution, sterile dextrose powder (Sigma-Aldrich, France) was added to sterile 0.9% NaCl solution and sonicated until homogenization in a 50 mL sterile tube. Only sham had free access to water during the experiment. In case of mortality before the end of infusion, a new envelope was added to the randomization box.

Three different experiment were realized in dedicated pools of rats: 1) echocardiography followed by gut laser speckle imaging and blood samples; 2) Evans Blue assay; 3) hemodynamic by left ventricle catheterization. These 3 sets of experiments necessitated different rats because of the incompatibility of Evans Blue or Hemodynamic with other assays.

Echocardiography

Transthoracic echocardiography was performed using a Vivid 7 ultrasound device (GE Healthcare, France) using a 11 Mhz linear probe. The heart was imaged with a two-dimensional parasternal short axis view. After visualization of the pillars of mitral valve, M-Mode imaging was performed measuring the left ventricular end-diastolic and end-systolic diameters (LVEDD and LVESD). Left ventricular fractional shortening (LVFS) was calculated as . Then, an apical view of the left ventricle was performed and a pulsed wave Doppler was used for acquisition of mitral peak velocity flow during diastole. The ratio between early (E) and late (A) peak velocities was calculated (E/A ratio). Proximal pulmonary artery (PA) was recorded for heart rate (HR), radius and velocity time integral (VTI), allowing the calculation of stroke volume ( and cardiac output (CO=SV x HR), expressed per gram of animal.


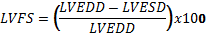

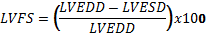

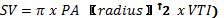

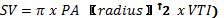


Analysis of mesenteric perfusion

After echocardiography, a wide median laparotomy was performed. The ileum was disposed in a similar manner in all animals and humidified with a 37°C warmed compress soaked with 0.9%NaCl. Microcirculation acquisition was performed by Laser Speckle Contrast Imaging at a 20 cm distance and a 1500 frames/256 colors acquisition (MoorLFPI-2 Full Field Laser Perfusion Imager – Moor Instruments, UK) five minutes after the laparotomy. Image analysis was performed in four similar regions of interest on the gut, distant from the jejunal site, and mean values are expressed as perfusion units (PU).

Capillary leakage and Evans Blue assay

Thirty mg/kg of 1% Evans blue (Sigma, France) was injected intravenously over a 2 min period. 45 min later, the animal was anesthetized and, after opening of the right auricula, a 18G cannula was inserted through the left ventricle into the ascending aorta to flush away intravascular dye from the bloodstream by 1% paraformaldehyde (PFA) perfusion in heparinized saline solution, at a pressure of 120 mmHg for 3 min. Heart (left ventricle), lungs, gut and liver samples were taken and dehydrated at 60°C for 5 days. The samples were incubated in a 10% solution of formamide (10 µL/mg of tissue, Sigma, France) at 37°C for 3 days and then centrifuged at 3000G for 15 min. The absorbance of the supernatant was measured using spectrophotometry at 620 nm.

Hemodynamics

Left-ventricular (LV) pressure-volume loops and hemodynamics were obtained using a miniaturized combined conductance catheter-micromanometer (model SPR-671, Millar Instruments, USA) connected to a pressure-conductance unit (MPCU-2000, Millar Instruments, USA), after insertion through the right carotid into the LV under pressure control. LV end-systolic pressure (LVESP), LV end-diastolic pressure (LVEDP), dP/dt_min_, dP/dt_max_ and LV relaxation constant Tau (Weiss Method) were recorded/calculated. Pressure-volume loops were recorded at baseline and during gently occlusion of abdominal aorta to calculate LVESP and LVEDP- relation (LVESPVR and LVEDPVR) calculated as indicators of load-independent LV contractile function and LV compliance. Blood pressure was measured immediately before crossing the aortic valve. At the end of the procedure, a blood sample was collected from the abdominal aorta into 4 mL ethylenediaminetetraacetic acid coated tube. Osmolality of the solution was measured using a cryoscopic osmometer and results are expressed in mosmol/kg of plasma.

Biological parameters

At the end of the procedures, a maximal volume of blood was sampled form the abdominal aorta into 4 mL-ethylenediaminetetraacetic acid coated tubes and then centrifuged at 1500 G for 15 min before immediate freezing in liquid nitrogen and storage at -80°C until assays.

Inflammation and capillary leakage related markers were analyzed on plasma samples using Enzyme Linked Immunosorbent Assay (ELISA) specific kits with the following sensitivity and detection ranges.

Interleukine-1𝛃: Sensitivity < 5 pg/mL. Detection range [31.3-2000 pg/mL]. Intra and inter assay variation <5%. RDsystem® kit RLB00

Tumor Necrosis Factor 𝛂: Sensitivity < 5 pg/mL. Detection range [12.5-800 pg/mL]. Intra and inter assay variation <10%. RDsystem® kit RTA00

Interleukine-10: Sensitivity < 10 pg/mL. Detection range [31.3-2000 pg/mL]. Intra and inter assay variation <10%. RDsystem® kit R1000

Vascular Endothelial Growth Factor type: Sensitivity 10 pg/mL. Detection range [62.5 – 2000 pg/mL], Intra and Inter assay variation < 15%, MyBioSource® kit MBS043103

Syndecan-1: Senstivity 1 pg/mL. Detection range [6.25 – 200 pg/mL], Intra and Inter assay variation < 15%, MyBioSource® kit MBS061601

*Biochemistery*: Urea, sodium, potassium, chloride and albumin were measured (Catalyst One, IDEXX, USA). Urine concentration of sodium, potassium and chloride were also measured, and absolute quantity of ions excreted during the experiment was calculated. Then, the difference between the infused and excreted amount of ion during the whole experiment was calculated as ∆*ion*=(*ion* fluid concentration)*(volume of infused fluid)-(*ion* urinary concentration)*(volume of urine) and reflected the body excess of ion over the infusion period. Urine was collected throughout the whole study using metabolic cages and rats have no access to water excepted for Sham group.

*Metabolism*: metabolization of lactate may take place through its oxidation into pyruvate, which in turn may proceed to either the oxidative pathway, the gluconeogenesis route or the production of ketone bodies. Glucose blood content was performed using portable meters (Accu-check^©^ performa, Roche, USA). Deproteinization of blood was performed before measurement of plasma concentration of lactate, pyruvate, 3-hydroxybutyrate and acetoacetate: perchloric acid was added to blood in a 1:1 ratio and then 3 rounds of centrifugation at 3500 G during 15 min were realized before frozen at -20°C.


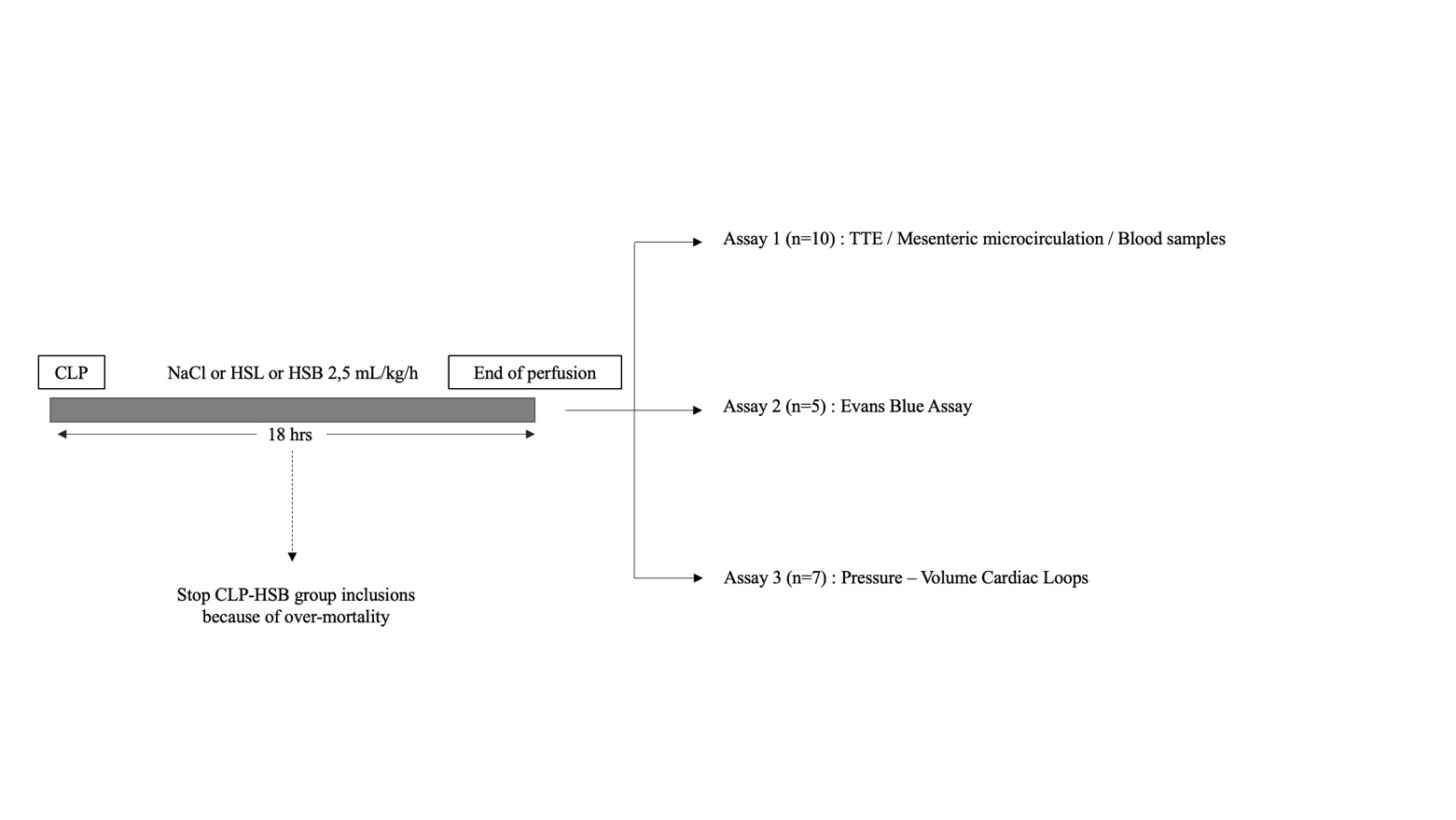


Schematic representation of the study design with the 3 different assays in the three completed groups (CLP-HSB interrupted before completion because of over-mortality)
